# Symptom management with osilodrostat in multiple endocrine neoplasia type 1 with a Cushing syndrome presentation

**DOI:** 10.1210/jcemcr/luag039

**Published:** 2026-03-31

**Authors:** Hasan Frookh Jamal

**Affiliations:** Department of Endocrinology, Government Hospital, Salmaniya Medical Complex, Manama 00973, Bahrain

**Keywords:** MEN1 syndrome, Cushing syndrome, osilodrostat, case study

## Abstract

Multiple endocrine neoplasia type 1 (MEN1) syndrome is a rare autosomal dominant disorder characterized by predisposition to a multitude of endocrine neoplasms. Cushing syndrome (CS) within MEN1 presents complex diagnostic and therapeutic challenges. We report a case of a 36-year-old male with adrenocorticotropic hormone (ACTH)-dependent Cushing disease and MEN1 syndrome with thymic and pancreatic neuroendocrine tumors. The patient presented with weight gain, weakness and proximal myopathy. Biochemical testing confirmed ACTH-dependent hypercortisolism. Imaging revealed a pituitary microadenoma, a large anterior mediastinal mass, and pancreatic lesions. Genetic analysis confirmed a pathogenic heterozygous MEN1 frameshift variant. After thymectomy, he declined further surgery. Treatment with thymectomy and octreotide long-acting release (LAR) was ineffective. Initiation of osilodrostat (titrated to 5 mg twice daily) resulted in a 76% reduction in urinary cortisol levels and a 47 kg weight loss, with significant functional improvement. Pancreatic and pituitary lesions remained stable. This is the first reported case of successful symptom management of MEN1-associated Cushing disease with osilodrostat, establishing it as an effective therapeutic option for medically complex MEN1 cases where surgery is not feasible or is declined.

## Introduction

Multiple endocrine neoplasia type 1 (MEN1) is a rare autosomal dominant disorder characterized by hyperparathyroidism and tumors of the duodenopancreatic neuroendocrine system and the anterior pituitary [1]. Adrenal involvement occurs in 20-73% of cases, but hormonal hypersecretion is uncommon [1]. Cushing syndrome (CS) in MEN1 can be adrenocorticotropic hormone (ACTH)-dependent, originating from either a pituitary adenoma (Cushing disease) or, more rarely, an ectopic ACTH-secreting tumor (thymic carcinoid), or ACTH-independent from an adrenal adenoma [2].

This complexity makes diagnosis and management challenging. We report a case of a patient with MEN1 syndrome presenting with ACTH-dependent Cushing disease, who declined definitive surgery and was subsequently managed successfully with the cortisol synthesis inhibitor, osilodrostat.

## Case presentation

A 36-year-old Bahraini male with a 2-year history of type 2 diabetes and hypertension presented with a 25 kg weight gain over 1 year, generalized weakness, shortness of breath, and proximal myopathy. At presentation, his blood pressure was 175/92 mmHg, serum potassium was 3.1 mmol/L, and HbA1c was 8.5%. Physical examination revealed central obesity, abdominal striae, a dorsocervical fat pad, multiple skin bruises, and proximal muscle weakness. His symptoms severely impacted his activities of daily living and ability to work.

## Diagnostic assessment

Initial biochemical testing confirmed ACTH-dependent Cushing disease: 2 elevated 24-hour urinary free cortisol (UFC) levels 1560 µg/24 hours (SI: 4305 nmol/24 hours); normal < 140 µg/24 hours (<386 nmol/24 hours), a nonsuppressed cortisol after a 1 mg overnight dexamethasone suppression test (6.4 µg/dL [SI: 178 nmol/L]; normal <1.8 µg/dL [SI: <50 nmol/L]), and an elevated ACTH level (208.9 pg/mL [SI: 46 pmol/L]; normal <45.4 pg/mL [SI: <10 pmol/L]). A low-dose DST was not performed, the strong clinical and biochemical evidence warranted direct localization with inferior petrosal sinus sampling (IPSS).

**Figure 1:**
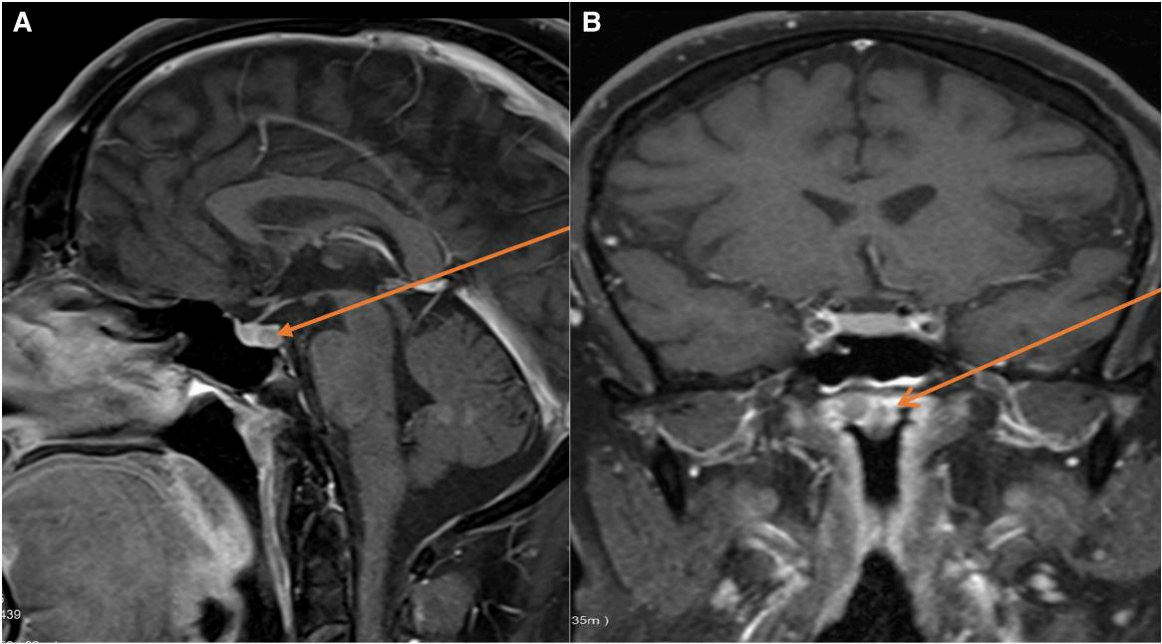
(A and B) Dynamic contrast-enhanced T1-weighted MRI of the pituitary gland demonstrating a small pituitary microadenoma. (A) Sagittal view. (B) Coronal view.

Pituitary magnetic resonance imaging (MRI) identified a 3 mm microadenoma (see Fig. 1). A computed tomography (CT) scan of the chest revealed a large 3.5 cm anterior mediastinal mass within the thymus. A Gallium-68 DOTATATE (Ga⁶⁸ DOTA-0-Tyr3-octreotate DOTATATE) positron emission tomography (PET)/CT scan revealed multiple somatostatin receptor-positive lesions: a 1 cm lesion in the pancreatic tail (maximum standardized uptake value [SUVmax] 27.9), a 1 cm lesion in the pancreatic body (SUVmax 50.2), and a 0.5 cm lesion in the pancreatic head (SUVmax 14.1). No significant uptake was noted in the anterior mediastinum post-thymectomy. The pancreatic lesions were not biopsied and were managed conservatively with surveillance.

The patient underwent a partial thymectomy. Histopathological analysis confirmed an atypical carcinoid tumor, characterized by a mitotic activity of 4 mitotic figures per 10 high-power fields (MF/10HPF), a Ki67 proliferation index of 5%, and a pathological stage of pT1aN1. Given the multi-organ involvement, MEN1 syndrome was suspected. Genetic analysis performed at Centogene GmbH (Rostock, Germany) confirmed a pathogenic heterozygous MEN1 frameshift pathogenic variant (c.202_206dup; p.Asp70Profs*51).

Baseline hormonal profiling revealed secondary hypogonadism (low luteinizing hormone, follicle-stimulating hormone, and testosterone) and vitamin D deficiency with secondary hyperparathyroidism (elevated parathyroid hormone with normal calcium). Thyroid function tests, insulin-like growth factor 1 and growth hormone were within normal limits (Table 1).

**Table 1:** Baseline hormonal and metabolic profile

Parameter	Value	Reference range	Date
ACTH	208.9 pg/mL (SI: 46 pmol/L)	< 45.4 pg/mL (SI: < 10 pmol/L)	Apr 2022
Basal cortisol 0800h	893 nmol/L (SI: 32.37 µg/dL)	140-690 nmol/L (SI: 10-20 µg/dL)	Apr 2022
24-hour urinary free cortisol	1560 µg/24 hours (SI: 4305 nmol/24 hours)	< 140 µg/24 hours (SI: <386 nmol/24 hours)	Apr 2022
HbA1c	8.5% (SI: 69 mmol/mol)	< 5.7% (SI: <39 mmol/mol)	Apr 2022
Potassium	3.1 mmol/L	3.5-5.2 mmol/L	Apr 2022
1 mg ODST	6.4 µg/dL (SI: 178 nmol/L)	< 1.8 µg/dL (SI: < 50 nmol/L)	Apr 2022
Calcium	9.1 mg/dL (SI: 2.27 mmol/L)	8.6-10.4 mg/dL (SI: 2.15-2.60 mmol/L)	Apr 2022
PTH	143.5 pg/mL (SI: 15.1 pmol/L)	18.6-88.6 pg/mL (SI: 1.96-9.33 pmol/L)	Apr 2022
Vitamin D	16.4 ng/mL (SI: 41 nmol/L)	>30 ng/mL (SI: >75 nmol/L)	Apr 2022
LH	1.2 IU/L	1.5-9.3 IU/L	Apr 2022
FSH	2.1 IU/L	1.6-11.0 IU/L	Apr 2022
Testosterone	61.2 ng/dL (SI: 2.12 nmol/L)	262.5-1154.0 ng/dL (SI: 9.1-40 nmol/L)	Apr 2022
Prolactin	14 ng/mL (SI: 297 mIU/L)	0.7-16.8 ng/mL (SI: 15-356 mIU/L)	Apr 2022
TSH	1.8 mIU/L	0.4-4.0 mIU/L	Apr 2022
Free T4	1.2 ng/dL (SI: 15.4 pmol/L)	0.8-1.8 ng/dL (10.3-23.2 pmol/L)	Apr 2022
IGF-1	145 ng/mL (SI: 145 µg/L)	101-267 ng/mL (101-267 µg/L)	Apr 2022
GH	2.1 µg/L (SI: 2.1 ng/mL)	0.4-10 µg/L (SI: 0.4-10 ng/mL)	Apr 2022

Abbreviations: ACTH, adrenocorticotropic hormone; FSH, follicle-stimulating hormone; GH, growth hormone; HbA1c, glycated hemoglobin; IGF1, insulin-like growth factor 1; LH, luteinizing hormone; ODST, overnight dexamethasone suppression test; PTH, parathyroid hormone; TSH, thyroid-stimulating hormone.

## Treatment

Post-thymectomy, the patient was started on octreotide long-acting release (LAR) at 20 mg (monthly) for its potential antiproliferative effect on the pancreatic neuroendocrine tumors and to control ACTH secretion from a suspected ectopic source [3]. However, hypercortisolism persisted, with UFC levels elevated at 1560 µg/24H (SI: 4306 nmol/24H) (normal <140 µg/24H [SI: <386 nmol/24H]). A follow-up Ga^68^ -DOTATATE scan 6 months later showed mild progression of the pancreatic lesions, with the body lesion now measuring 1.4 cm (SUVmax 76.2). The octreotide LAR dose was subsequently increased to 30 mg monthly. The patient declined any further surgical interventions. We opted for osilodrostat over other adrenal steroidogenesis inhibitors like metyrapone due to its potent 11β-hydroxylase inhibition, newer approval, and twice-daily dosing schedule, which may improve adherence, and because metyrapone was not commercially available in our country at the time. Osilodrostat was initiated at 2 mg twice daily and titrated to 5 mg twice daily.

## Outcome and follow-up

**Figure 2:**
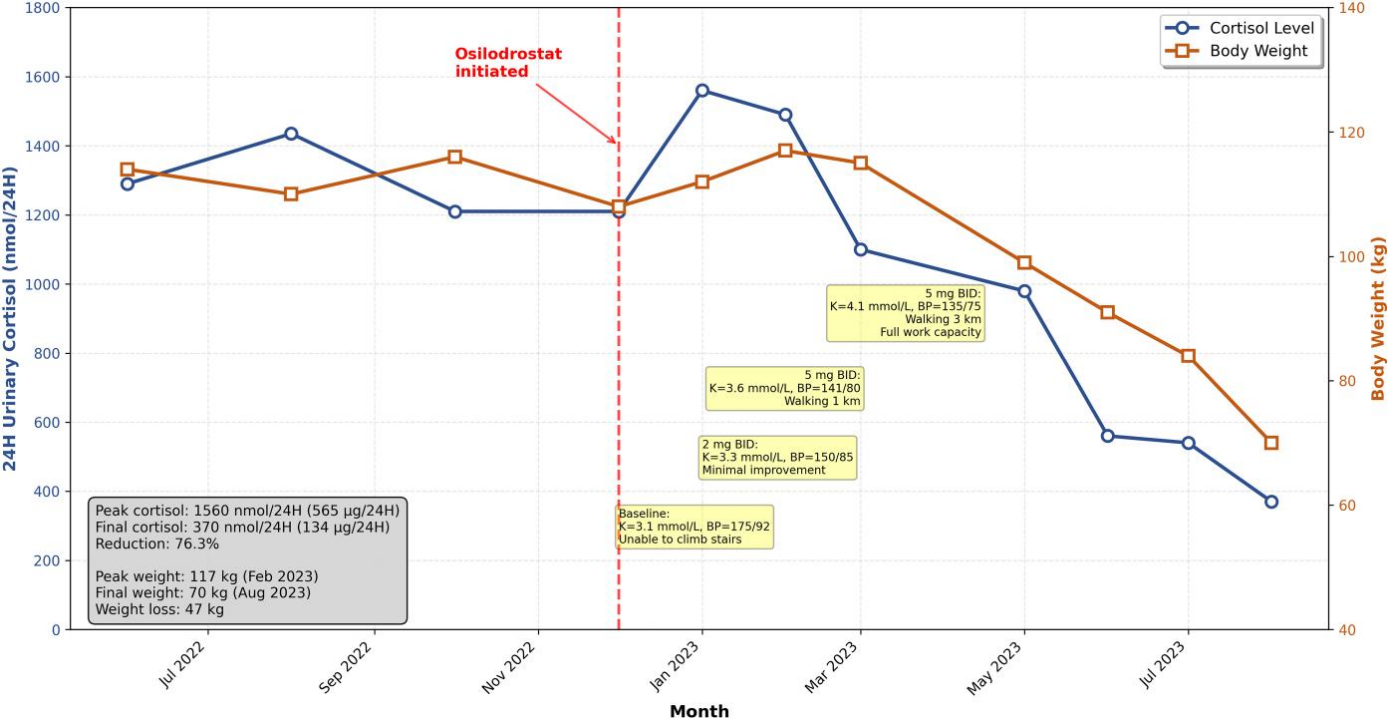
Changes in 24H urinary cortisol and body weight with osilodrostat treatment (including clinical response parameters). Conversion: nmol/24H ÷ 2.76 = µg/24H.

The response was dramatic. UFC levels were reduced by 76%, and the patient lost approximately 47 kg over 6 months (Fig. 2). This was accompanied by significant functional improvement, including the ability to walk 3 km daily and return to full-time work (Table 2). Pretreatment hypokalemia (3.1 mmol/L) and hypertension (175/92 mmHg) resolved without specific intervention, normalizing to 4.1 mmol/L and 135/75 mmHg, respectively (Table 2). The patient is monitored for potential adrenal insufficiency and is educated on sick-day rules.

**Table 2:** Osilodrostat response

Timepoint	Dose	K (mmol/L)	BP (mmHg)	Functional improvement
Baseline	None	3.1 mmol/L	175/92	Unable to climb stairs/pray
Week 4	2 mg BID	3.3 mmol/L	150/85	Minimal mobility improvement
Week 12	5 mg BID	3.6 mmol/L	141/80	Walking 1 Km, partial work return
Week 16+	5 mg BID	4.1 mmol/L	135/75	Walking 3 Km, full work capacity

Abbreviations: BID, *bis in die* (twice daily); BP, blood pressure

Osteoporosis was diagnosed by dual-energy X-ray absorptiometry (DXA) in April 2022, which revealed a T-score of −3.6 at the lumbar spine (L1-L4). The patient's current regimen therefore includes osilodrostat (5 mg twice daily [bid]), octreotide LAR (30 mg monthly), testosterone (250 mg intramuscular monthly), and denosumab (60 mg every 6 months) for bone protection. Follow-up imaging shows stable disease.

After a 6-week washout of osilodrostat, an IPSS procedure was performed, which confirmed a central pituitary source of ACTH (Table 3). Additionally, a corticotropin-releasing hormone (CRH) stimulation test was performed as part of the comprehensive diagnostic workup, the results of which were consistent with a pituitary etiology (Table 4).

**Table 3:** Inferior petrosal sinus sampling with desmopressin (DDAVP)

	Peripheral	Right petrosal	Left petrosal
Baseline	ACTH 465 pg/mL (SI: 102.3 pmol/L) Prolactin 39.5 ng/mL (SI: 39.5 µg/L)	ACTH 6637 pg/mL (SI: 1461.4 pmol/L) Prolactin 104.8 ng/mL (SI: 104.8 µg/L)	ACTH 813.7 pg/mL (SI: 179.2 pmol/L) Prolactin 96.76 ng/mL (SI: 96.8 µg/L)
Given DDAVP over 1 minute			
Post injection ACTH	442 pg/mL (SI: 97.3 pmol/L)	752.8 pg/mL (SI: 165.8 pmol/L)	1271 pg/mL (SI: 279.9 pmol/L)
2 minutes ACTH	437.3 pg/mL (SI: 96.3 pmol/L)	875.5 pg/mL (SI: 192.8 pmol/L)	1933 pg/mL (SI: 425.6 pmol/L)
5 minutes ACTH	452.4 pg/mL (SI: 99.6 pmol/L)	709.1 pg/mL (SI: 156.1 pmol/L)	1515 pg/mL (SI: 333.6 pmol/L)
10 minutes ACTH	406.2 pg/mL (SI: 89.4 pmol/L)	570.5 pg/mL (SI: 125.6 pmol/L)	1399 pg/mL (SI: 308.0 pmol/L)
Superior vena cava	478.1 pg/mL (SI: 105.3 pmol/L)		
Inferior vena cava	383.4 pg/mL (SI: 84.4 pmol/L)		

Abbreviations: ACTH, adrenocorticotropic hormone; DDAVP, desmopressin.

**Table 4:** Corticotrophin-releasing hormone stimulation test

Time	−15 minutes	0 minutes	15 minutes	30 minutes	45 minutes	60 minutes	90 minutes	120 minutes
Cortisol	15.63 µg/dL (SI: 431 nmol/L)	16.52 µg/dL (SI: 456 nmol/L)	23.67 µg/dL (SI: 654 nmol/L)	27.48 µg/dL (SI: 759 nmol/L)	28.89 µg/dL (SI: 798 nmol/L)	25.93 µg/dL (SI: 716 nmol/L)	22.47 µg/dL (SI: 620 nmol/L)	19.26 µg/dL (SI: 532 nmol/L)
ACTH		108 pg/mL (SI: 23.7 pmol/L)	135 pg/mL (SI: 29.8 pmol/L)	437 pg/mL (SI: 96.2 pmol/L)	212 pg/mL (SI: 46.8 pmol/L)	253 pg/mL (SI: 55.6 pmol/L)	236 pg/mL (SI: 52.1 pmol/L)	157 pg/mL (SI: 34.6 pmol/L)

Abbreviation: ACTH, adrenocorticotropic hormone.

Although pituitary surgery was offered, the patient declined and opted to resume medical therapy with osilodrostat.

## Discussion

This case illustrates the intricate diagnostic and therapeutic challenges posed by MEN1 syndrome when it presents with ACTH-dependent Cushing disease. Our patient exhibited a classic clinical picture of CS, but the subsequent discovery of multi-organ involvement, a pituitary microadenoma, a thymic carcinoid, and pancreatic neuroendocrine tumors, signaled a more complex etiology. The diagnosis of MEN1 was unequivocally confirmed by genetic analysis performed at Centogene GmbH, which identified a pathogenic heterozygous MEN1 frameshift variant.

While MEN1 is most commonly associated with parathyroid tumors [4], its presentation with CS represents a major diagnostic challenge due to the potential for ACTH secretion from pituitary, ectopic (typically thymic), or adrenal sources [2, 5, 6].

Pituitary tumors are present in about 40% of MEN1 patients, and up to 5-10% of such tumors secrete ACTH. Ectopic tumoral ACTH secretion has also been observed in MEN1, almost exclusively originating from thymic neuroendocrine tumors [2]. Thymic neuroendocrine tumors in MEN1 are extremely rare, often exhibit an aggressive course, are typically nonfunctional and carry a poor prognosis [7, 8].

This complexity is underscored by analyses of large MEN1 cohorts, where the precise etiology of CS can sometimes remain elusive [5]. As summarized in Table 5, which categorizes the etiologies of CS in reported MEN1 cases, several cases of functional ACTH-secreting thymic tumors in MEN1 have been reported [6, 8-13], highlighting this clinical scenario. This case exemplifies this diagnostic dilemma, where the co-existence of a pituitary microadenoma, thymic carcinoid and pancreatic neuroendocrine tumors necessitated extensive investigation [2]. In this context of diagnostic uncertainty, where an ectopic ACTH source from the thymic or pancreatic tumors was a strong possibility, a trial of octreotide LAR was initiated for its potential antiproliferative effect and to reduce ACTH secretion from a suspected ectopic source [3]. It was initially thought that the source of hypercortisolism was the thymus, as many MEN1 cases with CS present with ectopic ACTH secretion [6, 8-13]. However, in this case, the failure of thymectomy to correct the hypercortisolism was a pivotal clue that the primary source lay elsewhere.

**Table 5:** Etiology of Cushing syndrome in reported MEN1 cases

Source of CS	Number of cases (representative references)	Notes
Pituitary (Cushing disease)	70-80% [5, 14, 15]	Most common cause in larger cohorts
Adrenal	20-30% [5, 16]	Can be adenoma or carcinoma
Thymic	Rare <5% [7, 8, 10-13]	Often aggressive, carries poor prognosis
Unknown	Reported [5]	Etiology not definitively established

Abbreviations: CS, Cushing syndrome; MEN1, multiple endocrine neoplasia type 1.

The patient failed to respond biochemically to octreotide. The subsequent IPSS and CRH stimulation test (Tables 3 and 4) were therefore critical, as they definitively confirmed a pituitary source, classifying this as Cushing disease and explaining the lack of response to octreotide.

This case is noteworthy for 2 primary reasons. First, the patient's refusal of further surgery after thymectomy created a therapeutic impasse, compounded by the failure of medical therapy with octreotide LAR. Second, and most significantly, to the best of our knowledge, this represents the first reported case of successful long-term biochemical and clinical control of MEN1-associated Cushing disease using osilodrostat. While osilodrostat is established for Cushing disease, its successful application in the complex diagnostic and therapeutic landscape of MEN1 represents a novel clinical insight.

The response to osilodrostat was dramatic and sustained. Not only did it effectively achieve a 76% reduction in UFC levels (Fig. 2), but it also led to profound clinical improvement, including a 47 kg weight loss, resolution of hypokalemia and hypertension (Table 2), and a complete restoration of functional capacity and quality of life. This suggests that osilodrostat is a potent and viable therapeutic option for patients in whom surgical intervention is not feasible or is declined.

The efficacy of osilodrostat in this case can be attributed to its distinct mechanism of action. Osilodrostat is a potent oral inhibitor of 11β-hydroxylase, the enzyme responsible for the final step of cortisol synthesis in the adrenal gland [17]. By directly blocking adrenal cortisol production, it effectively controls hypercortisolism regardless of the ACTH source. This explains the rapid biochemical and clinical response observed in our patient.

While the response has been sustained and well-tolerated over 6 months, long-term vigilance is warranted. Potential challenges include escape from biochemical control due to compensatory rises in ACTH or the development of adverse effects such as hypoadrenalism, hypokalemia, or hypertension [18]. Furthermore, monitoring for potential growth of the pituitary microadenoma with periodic MRI is warranted. Ongoing monitoring of urinary free cortisol, electrolytes and blood pressure is also essential. This case adds to the growing evidence for osilodrostat's efficacy and supports its role as a viable long-term medical option for patients with Cushing disease who are not surgical candidates.

The case also underscores other management considerations in MEN1. Osteoporosis diagnosed by DXA scan justified the initiation of denosumab, highlighting the need for comprehensive bone health management. Furthermore, the persistent hypogonadism necessitated testosterone replacement, addressing another facet of this multi-system disorder.

In conclusion, navigating a concealed MEN1 syndrome manifesting as CS requires extensive imaging and genetic analysis. This case illustrates that in the presence of such complexity, a precise etiological diagnosis is paramount. Interventional procedures like IPSS remain the gold standard for differentiating between pituitary and ectopic ACTH sources. The initial lack of response to thymectomy and octreotide, followed by the definitive confirmation of a pituitary source, highlights this necessity. This case emphasizes that when surgery is not an option, osilodrostat can serve as an effective therapeutic pillar, offering complete clinical and biochemical control. This novel finding expands the treatment arsenal for this challenging patient population.

## Learning points

- First reported use of osilodrostat in MEN1-associated Cushing disease—achieved an approximate 76% reduction in urinary cortisol, 47 kg weight loss, and complete functional recovery when surgery was declined
- IPSS confirmed pituitary source despite concurrent thymic carcinoid—highlighting that multiple potential ACTH sources can coexist in MEN1, requiring systematic localization even after partial tumor resection
- Multi-modal therapy is required for comprehensive MEN1 management: osilodrostat for hypercortisolism, octreotide LAR for neuroendocrine tumors, testosterone replacement for hypogonadism, and denosumab for osteoporosis

## Data Availability

Original data generated and analyzed during this study are included in this published article.

## References

[1] Al-Salameh A, Cadiot G, Calender A, Goudet P, Chanson P. Clinical aspects of multiple endocrine neoplasia type 1. Nat Rev Endocrinol. 2021;17(4):207‐224. 33564173 10.1038/s41574-021-00468-3

[2] Simonds WF . Expressions of Cushing's syndrome in multiple endocrine neoplasia type 1. Front Endocrinol (Lausanne). 2023;14:1183297. 37409236 10.3389/fendo.2023.1183297 PMC10319112

[3] Zhang D, Lu L, Zhu HJ, et al Somatostatin treatment for ectopic ACTH syndrome due to pancreatic neuroendocrine tumors: review of the literature. Int J Endocrinol. 2022;2022:6283706. 35265125 10.1155/2022/6283706 PMC8901294

[4] Koyama N, Nagase T, Kure M, et al Multiple endocrine neoplasia type 1 with functional parathyroid cysts. Intern Med. 2022;61(8):1183‐1188. 34645755 10.2169/internalmedicine.7505-21 PMC9107977

[5] Simonds WF, Varghese S, Marx SJ, Nieman LK. Cushing's syndrome in multiple endocrine neoplasia type 1. Clin Endocrinol (Oxf). 2012;76(3):379‐386. 21916912 10.1111/j.1365-2265.2011.04220.x PMC3243821

[6] Al Brahim NY, Rambaldini G, Ezzat S, Asa SL. Complex endocrinopathies in MEN-1: diagnostic dilemmas in endocrine oncology. Endocr Pathol. 2007;18(1):37‐41. 17652799 10.1007/s12022-007-0008-6

[7] Hasani-Ranjbar S, Rahmanian M, Ebrahim-Habibi A, et al Ectopic Cushing syndrome associated with thymic carcinoid tumor as the first presentation of MEN1 syndrome-report of a family with MEN1 gene mutation. Fam Cancer. 2014;13(2):267‐272. 24218143 10.1007/s10689-013-9692-1

[8] Fujiwara W, Haruki T, Kidokoro Y, et al Cushing's syndrome caused by ACTH-producing thymic typical carcinoid with local invasion and regional lymph node metastasis: a case report. Surg Case Rep. 2018;4(1):55. 29892916 10.1186/s40792-018-0459-7 PMC5995764

[9] Jia R, Sulentic P, Xu JM, Grossman AB. Thymic neuroendocrine neoplasms: biological behaviour and therapy. Neuroendocrinology. 2017;105(2):105‐114. 28355610 10.1159/000472255

[10] Ghazi AA, Dezfooli AA, Mohamadi F, et al Cushing syndrome secondary to a thymic carcinoid tumor due to multiple endocrine neoplasia type 1. Endocr Pract. 2011;17(4):e92‐e96. 21550948 10.4158/EP11038.CR

[11] Li X, Su J, Zhao L, et al Familial Cushing syndrome due to thymic carcinoids in a multiple endocrine neoplasia type 1 kindred. Endocrine. 2014;47(1):183‐190. 24452869 10.1007/s12020-013-0141-6

[12] Takagi J, Otake K, Morishita M, et al Multiple endocrine neoplasia type I and Cushing's syndrome due to an aggressive ACTH producing thymic carcinoid. Intern Med. 2006;45(2):81‐86. 16484744 10.2169/internalmedicine.45.1427

[13] Asemota IR, Ajiboye O, Nwaichi C, Mbachi C, Mba B. Cushing's syndrome due to a functional thymic neuroendocrine tumor in multiple endocrine neoplasia type 1 syndrome. Cureus. 2021;13(10):e18590. 34760427 10.7759/cureus.18590 PMC8572323

[14] Matsuzaki LN, Canto-Costa MH, Hauache OM. Cushing's disease as the first clinical manifestation of multiple endocrine neoplasia type 1 (MEN1) associated with an R460X mutation of the MEN1 gene. Clin Endocrinol (Oxf). 2004;60(1):142‐143. 14678300 10.1111/j.1365-2265.2004.01943.x

[15] Rix M, Hertel NT, Nielsen FC, et al Cushing's disease in childhood as the first manifestation of multiple endocrine neoplasia syndrome type 1. Eur J Endocrinol. 2004;151(6):709‐715. 15588237 10.1530/eje.0.1510709

[16] Alzahrani AS, Al-Khaldi N, Shi Y, et al Diagnosis by serendipity: Cushing syndrome attributable to cortisol-producing adrenal adenoma as the initial manifestation of multiple endocrine neoplasia type 1 due to a rare splicing site MEN1 gene mutation. Endocr Pract. 2008;14(5):595‐602. 18753104 10.4158/EP.14.5.595

[17] Bertagna X, Pivonello R, Fleseriu M, et al LCI699, a potent 11β-hydroxylase inhibitor, normalizes urinary cortisol in patients with Cushing's disease: results from a multicenter, proof-of-concept study. J Clin Endocrinol Metab. 2014;99(4):1375‐1383. 24423285 10.1210/jc.2013-2117

[18] Fleseriu M, Biller BMK, Bertherat J, et al Long-term efficacy and safety of osilodrostat in Cushing's disease: final results from a phase II study with an optional extension phase (LINC 2). Pituitary. 2022;25(6):959‐970. 36219274 10.1007/s11102-022-01280-6 PMC9675663

